# ASSOCIATION BETWEEN LEISURE TIME AND COMMUTING PHYSICAL ACTIVITIES WITH HEART RATE VARIABILITY IN MALE ADOLESCENTS

**DOI:** 10.1590/1984-0462/;2017;35;3;00007

**Published:** 2017-07-31

**Authors:** Aline Cabral Palmeira, Breno Quintella Farah, Antônio Henrique Germano Soares, Bruno Remígio Cavalcante, Diego Giulliano Destro Christofaro, Mauro Virgílio Gomes de Barros, Raphael Mendes Ritti-Dias

**Affiliations:** a Universidade de Pernambuco, Camaragibe, PE, Brasil.; b UPE, Recife, PE, Brasil.; c Universidade Estadual Paulista “Júlio de Mesquita Filho”, Presidente Prudente, SP, Brasil.; d Hospital Israelita Albert Einstein, São Paulo, SP, Brasil.

**Keywords:** Physical activity, Autonomic nervous system, Adolescent

## Abstract

**Objective:**

To investigate the association between heart rate variability (HRV) parameters with leisure time and commuting physical activities in adolescent boys.

**Methods:**

The sample included 1152 male adolescents aged 14 to 19 years. The variation of consecutive heart beats (RR intervals) was assessed and HRV parameters in time (SDNN, RMSSD, pNN50) and frequency domains (LF/HF) were calculated. Leisure time and commuting physical activities were obtained using a questionnaire. A binary logistic regression was performed between HRV parameters and physical activity.

**Results:**

Leisure time physical activity was associated with SDNN, RMSSD, pNN50, while LF/HF was not associated. These associations were stronger when adolescents were also physically active for more than six months. Commuting physical activity was not associated with any HRV parameter. Boys who practiced commuting physical activity and were also physically active for more than six months presented a lower chance of having low SDNN and RMSSD.

**Conclusions:**

Leisure time physical activity was associated with better HRV and these associations were enhanced when adolescents were physically active for more than six months. Commuting physical activity was not associated with HRV parameters; however, it became associated with better HRV when adolescents were physically active in commuting for more than six months.

## RESUMO

**Objetivo:**

Investigar a associação entre parâmetros de variabilidade da frequência cardíaca (VFC) e atividades físicas de lazer e deslocamento em adolescentes do sexo masculino.

**Métodos:**

A amostra incluiu 1.152 adolescentes do sexo masculino com idades entre 14 e 19 anos. A variação dos batimentos cardíacos consecutivos (intervalos entre duas ondas R sucessivas - RR) foi avaliada, e calcularam-se os parâmetros da VFC no tempo (desvio padrão de todos os intervalos RR - SDNN, raiz quadrada da média do quadrado das diferenças entre intervalos RR normais adjacentes - RMSSD, porcentagem dos intervalos RR adjacentes com diferença de duração maior que 50 ms - pNN50) e domínios de frequência (*low frequency* - *LF/high frequency* - *HF*). Informações sobre atividades físicas de lazer e deslocamento foram obtidas por meio de um questionário. Realizou-se regressão logística binária entre parâmetros de VFC e atividade física.

**Resultados:**

Foi descoberta associação entre atividades físicas de lazer e as variáveis SDNN, RMSSD e pNN50, mas não houve associação entre tais atividades e a razão LF/HF. Essas associações foram mais fortes entre adolescentes que se mantinham fisicamente ativos havia mais de seis meses. Atividades físicas de deslocamento não foram associadas a nenhum parâmetro de VFC. Jovens que praticavam atividades físicas de deslocamento e também se mantinham fisicamente ativos havia mais de seis meses apresentaram menor chance de ter baixa SDNN e RMSSD.

**Conclusões:**

Atividades físicas de lazer e de deslocamento foram associadas a melhor VFC, e tais associações foram reforçadas quando os adolescentes mantinham atividade física havia mais de seis meses. Atividade física de deslocamento não foi associada com os parâmetros da VFC, no entanto tal associação surgiu nos casos de adolescentes fisicamente ativos em atividades de deslocamento havia mais de seis meses.

## INTRODUCTION

Heart rate variability (HRV), which is defined as the variation of consecutive heartbeats, is a marker of cardiac autonomic modulation function. The autonomic nervous system promotes the interaction of the sympathetic pathways, which increase heart rate, contraction force, and vasoconstriction, with the parasympathetic pathways, which have opposite effects.^1^ Low HRV is an independent predictor of mortality and incidence of cardiovascular disease in adults.^2^ In children and adolescents, low HRV is associated with higher blood pressure levels^3^ and abdominal obesity,^4^ thus indicating its potential as a tool to screen cardiovascular risk among young people.

An increase in physical activity level has proven to be an effective way to increase HRV parameters in different age groups. In adolescents, although studies have shown a positive association between leisure-time physical activity and better HRV,^5^ ^,^ ^6^ ^,^ ^7^ ^,^ ^8^ there are still some gaps. For example, the association between commuting physical activity (walking or cycling to school), which is a common form of physical activity among adolescents, and HRV parameters is unknown.

Adolescent boys have an increased risk for earlier development of cardiovascular disease than girls.^9^ The HRV responses can provide subsidies for the implementation of health programs to improve cardiovascular health. This study’s hypothesis is that adolescents who engage in leisure-time and commuting physical activities would have better HRV than their inactive peers and the practice of physical activity for more than six months would bring a positive influence to HRV. Thus, the aim of this study was to investigate the association between the HRV parameters with leisure-time and commuting physical activities in adolescents and the influence of physical activity for more than six months on HRV.

## METHOD

This cross-sectional study was approved by the ethical committee in compliance with the Brazilian National Research Ethics System Guidelines. The participants were sampled from among students in the public school system in the state of Pernambuco in northeast Brazil.

The sample was selected by the application of a stratified two-stage (school and class) random sampling procedure. The schools were selected according to size (small, medium, and large) and the classes according to the shift (day or night). The draws were conducted by random number generation through www.randomizer.org site. Participants who were eligible for the study included male adolescents between the ages of 14 and 19 years who were in the classroom on the collection day and provided prior parental or guardian consent. Volunteers with known diabetes mellitus, cardiovascular disease, and neurological or mental disabilities were excluded, as well as those with a low-quality HRV signal or poorly completed questionnaire. The exclusion criteria also included consumption of caffeinated beverages 12 hours prior to the HRV evaluation, the use of alcohol, any form of tobacco and/or other illicit drugs, and participation in any physical exercise training 24 hours before the evaluations.^10^

Data were collected from May to October 2011 during the volunteers’ class period (morning, afternoon, and evening). To obtain data on physical activity level, age, ethnicity, housing area (rural or urban), and issues related to their economic condition, an adapted version of the Global School-based Student Health Survey (GSSHS) was used. This questionnaire has been widely applied in epidemiological studies with adolescents and has a reported concordance coefficient (kappa test) between 0.52 and 1.00.^3^ ^,^ ^11^ For this sample, leisure-time physical activity, commuting activity, and practice time presented a reproducibility indicator (i.e., test-retest consistency 1-week apart) respectively of 0.63, 0.59, and 0.57.

Leisure-time physical activity was assessed by the question: “Do you regularly perform some sort of physical activity in your free time, such as exercise, sports, dance or martial arts?” The adolescents were classified as active (if the answer was yes) or not active. Commuting physical activity was assessed by the question: ‘During the past seven days, how many days have you walked or cycled to and from school?’ The adolescents who responded that they got to and from school on foot or by bicycle three days or more were considered active. The time of regular practice of physical activity (i.e. physical activity for more than six months; PA>6 months) was assessed with the question “A young man is considered to be physically active if he engages in at least 60 minutes of daily physical activity on five or more days of the week. In relation to your physical activity habits, you say…” Adolescents were considered PA>6 months if they answered “I have been physically active for more than 6 months.”

Adolescents were weighed without shoes and coats on an automatic scale, and the height was measured with a stadiometer. Waist circumference was measured in the standing position at the level of the umbilicus using a constant tension tape. Overweight was determined by a body mass index measurement above the 85^th^ percentile for age.^12^ Abdominal obesity was determined by waist circumference above the 80th percentile for age.^13^ Blood pressure was measured by using the Omron HEM 742^14^ (Omron, Shangai, China) after the adolescents rested and remained seated with legs uncrossed for 5 minutes. All blood pressure measurements were performed 3 times on the right arm, which was placed at heart level in a seated position. The mean value of the least two measurements was used for analysis. High blood pressure was defined as systolic and/or diastolic blood pressure equal or higher than the reference sex, age, and height-specific 95^th^ percentile.^15^

Heart rate variability was obtained by analysis of RR intervals by using a heart rate monitor (POLAR, RS 800 CX; Polar Electro OyInc, Kempele, Finland). Adolescents were in a supine position for 10 minutes after approximately 30 minutes of rest. Analyses were performed using Kubios HRV software (Biosignal Analysis and Medical Imaging Group, Joensuu, Finland) by a single evaluator blind to the other variables and following the recommendations of the Task Force of the European Society of Cardiology and the North American Society of Pacing and Electrophysiology.^16^

The time domain variables consisted of the standard deviation of all RR intervals (SDNN), the root mean square of the squared differences between adjacent normal RR intervals (RMSSD), and the percentage of adjacent intervals over 50 ms (pNN50). The frequency domain was analyzed using the spectral analysis of HRV. Stationary periods of the tachogram of at least five minutes were broken down into bands of low (LF) and high (HF) frequencies by using the autoregressive method with a model order of 12 according to Akaike’s information criterion. Frequencies were considered physiologically significant when they ranged between 0.04 and 0.4 Hz. Oscillations between 0.04 and 0.15 Hz were considered as an LF component, whereas oscillations between 0.15 and 0.4 Hz represented the HF component. For analysis, we used the LF/HF component as an indicator of the sympathovagal balance on the heart. The reliability of the measurements was assessed using the intraclass correlation coefficient (ICC) and the values ranged from 0.70 to 0.91.^17^

The HRV parameters were classified in quartiles and then dichotomized for analysis (1^st^ quartile *versus* 2^nd^ to 4^th^ for SDNN, RMSSD, pNN50 and 4^th^ quartile *versus* 1^st^ to 3^rd^ quartiles for LF/HF). For the SDNN, RMSSD e pNN50 parameters, the 1^st^ quartile was considered the best and, for LF/HF parameters, the 4^th^ quartile was considered optimal.

All statistical analyses were performed by using the software SPSS/PASW version 20.0 (IBM Corp, Armonk, New York). Data normality was verified by the Kolmogorov-Smirnov test. The HRV parameters data are presented in mean, standard deviation, and confidence intervals (CI) of 95%.

The binary logistic regression analysis was performed to analyze the association between the leisure-time and commuting physical activities with HRV. A cluster analysis was also conducted and included a binary logistic regression between the parameters of HRV with leisure-time physical activity, along with the regular practice of physical activity (LPA+PA>6) and commuting physical activity along with regular practice of physical activity (CPA+PA>6). These findings were adjusted for the period of the day, obesity, and hypertension. The Hosmer-Lemeshow test was used to evaluate the goodness of fit. The significance level for all analysis was *p*<0.05.

## RESULTS

A total of 1212 male adolescents were enrolled in the study; 60 boys were excluded due to low signal quality (stationary periods of the tachogram lengths lower than 5 minutes). Thus, the final analysis consists of data from 1152 male adolescents with a mean age of 16.6±1.2 years. When asked which leisure activity they preferred, 43.8% of adolescents said playing sports. Table 1 describes the characteristics of the sample and the percentage of activity in each type of physical activity. Table 2 presents the criteria used for the stratification of HRV parameters in statistical analysis.

**Table 1:**
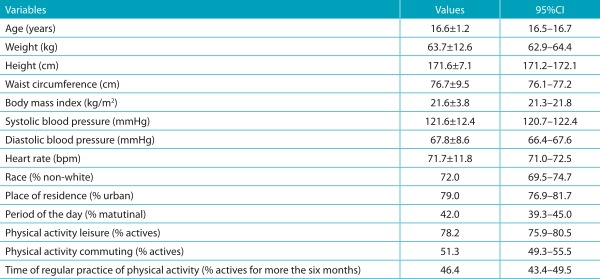
Characteristics of the studied adolescents (n=1152).

CI: confidence interval.

**Table 2:**
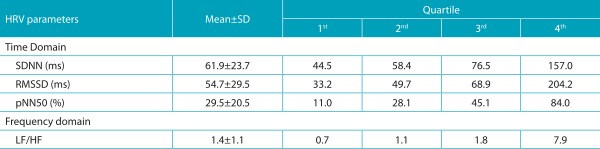
Criterion used for stratification of heart rate variability parameters in statistical analysis (n=1152).

SD: standard deviation; SDNN: standard deviation of the RR interval; RMSSD: root mean square of the squared differences between adjacent normal RR intervals; pNN50: the percentage of adjacent intervals over 50 ms; LF/HF: sympathovagal balance; HRV: heart rate variability.

**Figure 1:**
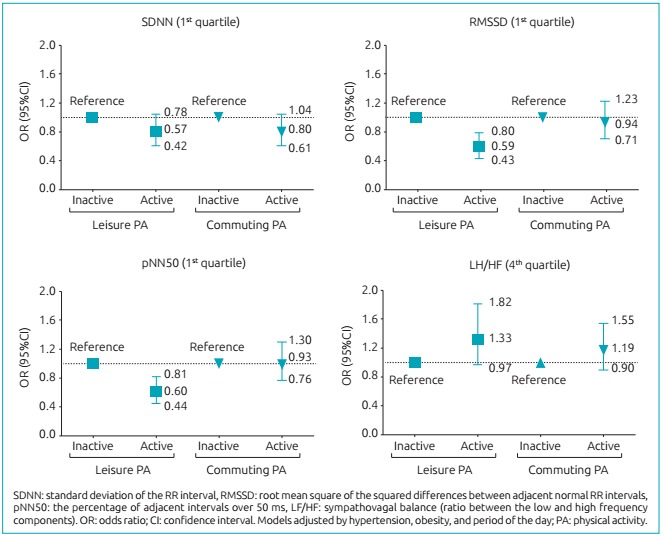
Association between the types of physical activity and the heart rate variability parameters.

The association between leisure-time and commuting physical activities with HRV parameters are shown in Figure 1. There were significant associations between leisure-time physical activity and HRV parameters in the time domain (SDNN (OR 0.57; 95%CI 0.42-0.78), RMSSD (OR 0.59; 95%CI 0.43-0.80), pNN50 (OR 0.60; 95%CI 0.44-0.81)). Commuting physical activity was not associated with any HRV parameter. The total volume of commuting physical activity was not associated with any HRV parameter (SDNN (*p*=0.937), RMSSD (*p*=0.664), pNN50 (*p*=0.323)).

The cluster of leisure-time physical activity and PA>6 months (Table 3) revealed significant associations with HRV in the time domain (*p*<0.05). The cluster of commuting physical activity and PA>6 months was significant (*p*<0.05) only in the SDNN and RMSSD parameters.

**Table 3:**
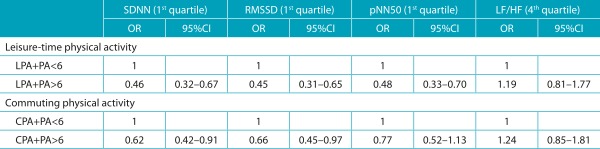
Association between the cluster the types of physical activity and heart rate variability parameters.

LPA: leisure-time physical activity; PA: physical activity; CPA: commuting physical activity. Models adjusted by hypertension, obesity and period of the day.

## DISCUSSION

The main results of this study were that:

1. leisure-time physical activity is associated with higher HRV;
2. the associations between leisure-time physical activity were enhanced when physical activity is practiced for more than 6 months;
3. commuting physical activity is not associated with HRV in adolescents;
4. however, the adolescent active in commuting physical activity for more than six months had higher HRV.

The strengths of this study include its large sample size, since there are no epidemiological studies with a sample of this size that analyzes HRV. We assessed blood pressure and obesity, which are important confounding variables and closely linked to HRV.^3^ ^,^ ^18^ Moreover, because the HRV was analyzed by only one investigator blind to all other study variables, the results are highly reproducible and reliable. Finally, the study examined the relationship of HRV and different physical activity, which has not been done previously.

Sports practice is the main frequent form of leisure-time physical activity among adolescent boys.^19^ The association between leisure-time physical activity and cardiovascular function,^20^ ^,^ ^21^ including HRV parameters, was previously described in adolescents.^7^ In this study, adolescents active during their leisure-time also presented better HRV. However, this study expands the current knowledge by indicating that adolescents active during leisure-time and who practiced physical activity for more than six months presented an even better HRV, suggesting that the accumulation of leisure-time physical activity has a positive impact on cardiovascular health in boys.

Although the mechanisms responsible for causing such effects are not clear, it is believed that regular physical activity brings changes that are evident at rest, such as a decrease in HR, improvements in the neuro-hormonal modulation caused by the decrease in catecholamine levels^22^ and angiotensin II.^22^ ^,^ ^23^ The practice of physical activity is related to the modulation of cardiorespiratory capacity, which may slow the reduction of parasympathetic activity.^21^ ^,^ ^24^ ^,^ ^25^ These adaptations alter the autonomic nervous system, increasing vagal tone and decreasing sympathetic activity in the heart.^26^ ^,^ ^27^

Commuting activity, such as walking or cycling, was not associated with HRV parameters and the main hypothesis for this outcome is related to the intensity of these activities. Active commuting is an option primarily when destinations are no more than 20 minutes^26^ ^,^ ^28^ and generally performed at a low-to-moderate intensity. Therefore, the intensity of commuting physical activities is probably not enough to promote adaptations in cardiac autonomic modulation.^3^ ^,^ ^20^ ^,^ ^29^ ^,^ ^30^ ^,^ ^31^ Interestingly, the commuting physical activity, along with the PA>6 months, was associated with the HRV parameters, which indicates that, for a long period, this kind of activity seems to be a beneficial influence to the cardiovascular system.

Some limitations of this study should be considered. The cross-sectional design is the main concern, as it limits the establishment of causal relationships. The sample included only male adolescents, and extrapolation for female adolescents is limited. Although the ages of participants were tightly controlled, we could not determine the Tanner stage of the participants. Thus, possible influences of biological maturation on the results were not controlled.^6^ The physical activity level was assessed subjectively by a questionnaire. Although the questionnaire is an indirect measure, it has a good reproducibility and allows for measuring the level of physical activity in different domains (leisure, commuting), which is not possible with direct measures such as pedometer and accelerometer are applied.^32^ The leisure-time physical activity included all leisurely physical activities and it was not possible to determine the influence of their different types. Also, commuting physical activity considered only the route to and from the school, without assessing other commuting physical activities.

It can be concluded that leisure-time physical active adolescents presented better HRV indices. The parameters of HRV presented better indices in adolescents who were active for more than six months in commuting physical activity.
